# Estimating statistical power, posterior probability and publication bias of psychological research using the observed replication rate

**DOI:** 10.1098/rsos.181190

**Published:** 2018-09-12

**Authors:** Michael Ingre, Gustav Nilsonne

**Affiliations:** 1 Department of Clinical Neuroscience, Karolinska Institutet, Solna, Sweden; 2 Institute for Globally Distributed Open Research and Education (IGDORE), Sweden; 3 Stress Research Institute, Stockholm University, Stockholm, Sweden; 4 Department of Psychology, Stanford University, Stanford, CA 94305, USA

**Keywords:** selection bias, prior probability, falsification

## Abstract

In this paper, we show how Bayes' theorem can be used to better understand the implications of the 36% reproducibility rate of published psychological findings reported by the Open Science Collaboration. We demonstrate a method to assess publication bias and show that the observed reproducibility rate was not consistent with an unbiased literature. We estimate a plausible range for the prior probability of this body of research, suggesting expected statistical power in the original studies of 48–75%, producing (positive) findings that were expected to be true 41–62% of the time. Publication bias was large, assuming a literature with 90% positive findings, indicating that negative evidence was expected to have been observed 55–98 times before one negative result was published. These findings imply that even when studied associations are truly NULL, we expect the literature to be dominated by statistically significant findings.

## 1. Introduction

The Open Science Collaboration (OSC) reported that 36% of published positive findings in experimental psychology were successfully replicated in independent attempts [1]. This finding is interesting in itself as an indicator of the reproducibility of published findings in psychology; however, it is also an important data point that can be used together with other information to assess publication bias, statistical power and even the posterior probability of findings published in the psychological literature.

Another important set of observations concern the proportion of positive findings in the literature. A series of observations spanning five decades have indicated that more than 90% of published studies in psychology reported positive findings, where the authors’ hypothesis was supported by data [2–4]. A similar observation was made by the OSC, where 97% of the original studies they replicated supported the proposed hypothesis with a ‘statistically significant’ association [1].

There are many sources of bias in research. In the following analysis, we take advantage of the fact that the OSC performed *direct replications* of the original studies, where the design, methods, materials, study population and statistical analysis of the result were reproduced as close to the original studies as possible. This means that many methodological biases have been controlled and cannot explain differences in the outcome between original studies and replications.

A large class of biases related to the process of publishing was not accounted for by the replications. In the replications, only one test of the hypothesis was performed and the finding was reported regardless of the result; however, the original studies had to make it through peer-review and were subject to editorial policies that have been suggested to favour novel and positive findings [5], creating selection bias in the published literature. Knowledge of this bias may also have caused researchers to adapt their strategy when observing a negative result: they may have put negative findings in the file drawer and looked for positive results in another study, or they may have tried to repeatedly observe different results in the same study until they found one that was positive. The first strategy creates bias that is generally known as the file drawer problem [6], and the latter is usually referred to as selective reporting, HARKing [7] or *p*-hacking [8–10]. They all produce a similar selection bias where observed negative evidence is suppressed in favour of reporting positive findings. We can estimate the collective magnitude of this *publication bias* by comparing the observed proportion of positive findings in the published literature, with the proportion of positive findings that was expected after a single test in the original studies that were replicated by the OSC.

In this paper, we show how the observed reproducibility and proportion of positive findings in the literature can be used to better understand meta-properties of published psychological research. We demonstrate a mathematical solution that can be used to assess *expected* statistical power, posterior probability and publication bias of published psychological research. We aim to answer the following questions:

— What are the properties of research that leads to 90% positive findings?
— What is the expected reproducibility of research with 90% positive findings?
— Is the observed 36% reproducibility rate consistent with an unbiased literature?
— What does the observed reproducibility suggest about the prior probability of the tested hypotheses, statistical power of the studies, the posterior probability of the original findings and of publication bias?

In the first part of our analysis, we use a naive approach. This analysis produces simple linear equations that are valid for a single study; but when they are applied to a group of studies in the literature, they assume that all studies have identical statistical power and tend to produce biased estimates when there is large variance in statistical power. However, when statistical power is assumed to be very high (i.e. more than 90%), there is little room for variance in power to influence the result, and the naive calculations approximate more complex solutions. The second part of our analysis takes variance in statistical power between studies into account, in order to produce more ecological estimates of the published literature.

The mathematical exercises presented here were performed in R [11], and the source code needed to reproduce all findings is available as the electronic supplementary material, appendix and on GitHub (https://github.com/micing/publication_bias_psychology).

## 2. What are the properties of research that leads to 90% positive findings?

The concept of *prior probability* from Bayesian theory [12] describes the probability that a hypothesis is true before it has been tested on data. When considering a large number of hypotheses, prior probability can also be understood as the proportion of hypotheses that are true *a priori*, that is, before they have been tested on data. The prior probability of an individual hypothesis can be small and close to zero, for example, in massively exploratory studies where vast amounts of data are searched to try to find the few true associations that may exist; or it can be large and close to one, in theoretically motivated confirmatory research with prior empirical support. We will use theta (*θ*) to denote *prior probability*.

We also need to consider the probability that a study testing a *true* hypothesis will produce positive evidence. This is generally known as statistical power within a NULL hypothesis significance testing (NHST) paradigm and is calculated from the type 2 error rate: 1 − *β*. Finally, we need to consider the test's type 1 error rate that describes the probability of observing positive evidence when the hypothesis is false, which we will assume to be *α* = 0.05 in this text unless stated otherwise.

The probability of observing true-positive evidence is calculated by multiplying the prior probability with the statistical power of the study (equation (2.1)). The probability to observe false-positive evidence is the type 1 error rate multiplied by the prior probability that the hypothesis is *false* (equation (2.2)). Added together, they describe the total probability of observing positive evidence (equation (2.3)).

2.1 $$P_{\mathrm{true}}\;=\theta (1-\beta ),$$

2.2 $$P_{\mathrm{false}}=\alpha (1-\theta )$$

2.3 $$\;\text{and}\;P_{\mathrm{total}}=\theta (1-\beta )+\alpha (1-\theta ).$$

If a hypothesis is true *a priori*, we cannot observe false-positive evidence, and the probability of observing positive evidence reduces to the statistical power (1 − *β*). This shows that one way to produce 90% positive findings is to only test true hypotheses with 90% power. Another way to produce close to 90% positive findings is to run studies with perfect power (100%) on hypotheses of which 90% are true *a priori*. It should be noted that in such a situation we would actually expect to observe 90.5% positive evidence, because we would also observe a small number of type 1 errors when the hypothesis is false, as described by equation (2.2). The smallest prior that can produce 90% expected positive evidence is 89.5%, assuming perfect power. Thus, it is possible to produce an unbiased literature with more than 90% positive findings when the underlying research tests hypotheses that are more than 90% true *a priori* in studies with more than 90% statistical power.

## 3. What is the expected reproducibility of research with 90% positive findings?

To calculate the reproducibility of a positive research finding, we first need to calculate the probability of such a finding to be true (rather than a type 1 error). We can do this by applying Bayes' theorem [12] in order to calculate the *posterior probability*:

3.1 $$P(A|B)=\frac{P(B|A)P(A)}{P(B)}.$$

In this equation, we calculate the conditional probability of *A* given *B*. If we replace *A* with a hypothesis, and *B* with observing positive evidence, we can calculate the posterior probability of a hypothesis given that we have observed positive evidence. The numerator then describes the probability of observing positive evidence given that the hypothesis is true, which is statistical power, multiplied by the prior probability of the hypothesis; and this is precisely *P*_true_ that we defined earlier in equation (2.1). The denominator is the total probability of observing positive evidence, which is *P*_total_ defined by equation (2.3). Thus, we merely need to take the ratio *P*_true_/*P*_total_ defined by equations (2.1)–(2.3), to complete a formulation of Bayes’ theorem that can be used to estimate the *posterior probability* of a hypothesis after observing positive evidence from NHST:

3.2 $$\hat{\theta }=\;\frac{\theta (1-\beta )}{\theta (1-\beta )+\alpha (1-\theta )}.$$

When we know the posterior probability and statistical power, it is easy to calculate the probability of a positive finding to be reproduced (*R*) in an identical independent study. Equation (2.3) above already showed how to calculate the probability of observing positive evidence, but in this case we substitute the assumed prior probability (*θ*) with the posterior probability ($\hat{\theta }$) of the finding:

3.3 $$R=\hat{\theta }(1-\beta )+\;\alpha (1-\hat{\theta }).$$

As discussed above, with a perfect prior and 90% power, we would observe 90% positive findings that are all true; the reproducibility of such a finding in an identical study is the same as the statistical power 90%. At the other end of the spectrum, we find the smallest prior able to produce 90% expected positive evidence at 89.5%, assuming perfect power; and applying equations (3.2) and (3.3) indicates a posterior probability and reproducibility of such research at 99.4%. Thus, the expected reproducibility of research producing more than 90% positive evidence falls in the range of 90–100%.

## 4. Is the observed 36% reproducibility consistent with an unbiased literature?

We can use the above information to create a tentative statistical test of bias of the published literature. A binomial test on the observed reproducibility rate of 36% (95% CI 27–46%; *n* = 97) reported by the OSC indicates strong evidence (*p* < 10^−15^) that the replication studies were not drawn from a literature with 90% reproducibility. This conservative test, assuming the lower bound of reproducibility that is expected in an unbiased literature with 90% positive evidence, and identical power in the replication studies, indicates publication bias in the OSC sample, supporting the observation made in the original report of a right-skewed funnel plot [1].

## 5. Incorporating reproducibility into Bayesian calculations

One complication with applying Bayes' theorem (equation (3.2)) is that it is based on several unknown variables. We usually have a good idea of the type 1 error rate that is applied in research, but prior probability and statistical power are often elusive. We can sometimes make informed guesses [13] and calculate the posterior probability, as illustrated above, but with three unknown variables, statistical power (1 − *β*), prior (*θ*) and posterior ($\hat{\theta }$), there is only a limited amount of information we can extract from data. We want to reduce the number of unknown variables to only two, so that we can learn more useful information.

A first step in this process is to form a system of equations based on equations (3.2) and (3.3), so that we can incorporate the observed reproducibility into our calculations (equation (5.1)):

5.1 $$\left\{ \begin{array}{rl} \hat{\theta } & =\;\frac{\theta (1-\beta )}{\theta (1-\beta )+\alpha (1-\theta )}\\ R & =\hat{\theta }(1-\beta )+\;\alpha (1-\hat{\theta }). \end{array}\right.$$

If we knew the type 1 error rate (*α*) and the probability of a positive finding to be reproduced in an identical study (*R*), equation (5.1) would have only two unknowns (*β* and *θ*) and we could solve it to find the statistical power (1 − *β*) needed for any assumed prior (*θ*).

## 6. Accounting for variance in statistical power

So far, we have used a naive approach that is valid for a single hypothesis tested in identical studies, but when applied to a group of studies published in the literature, it assumes that all studies have identical statistical power, which is not plausible in general. Equations (6.1) and (6.2) below take variance into account by integrating the result over a probability density function (*f*) with mean *μ*_β_, describing the distribution of statistical power (1 − *β*) between studies. Assuming that we know the type 1 error rate (*α*) and prior probability (*θ*) of the research, these equations produce the *expected* posterior probability (equation (6.1)) and the *expected* reproducibility (equation (6.2)) of the research; the complement of the mean of *f* is also the *expected* statistical power (1 − *μ*_β_):

6.1 $$\text{E}[\hat{\theta }]=\;\int_{0}^{1}\frac{\;f(\beta )\theta (1-\beta )}{\theta (1-\beta )+\alpha (1-\theta )}\text{d}\beta$$

and

6.2 $$\text{E}[R]=\text{E}[\hat{\theta }](1-\beta )+\;\alpha (1-\text{E}[\hat{\theta }]).$$

**Figure 1.**
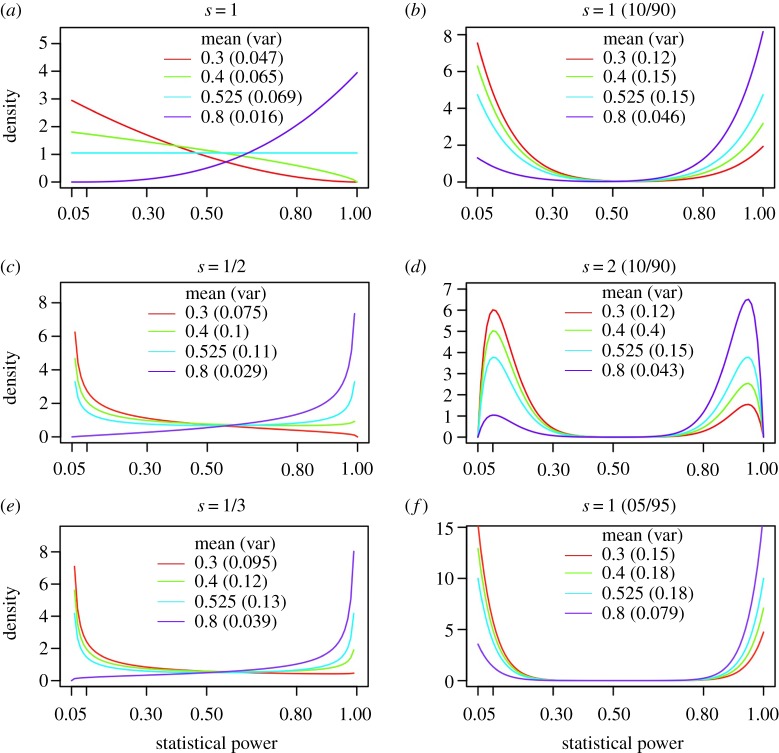
Beta distributions used to model variance in statistical power. (*a*,*c*,*e*) Beta distributions defined by a single shape parameter, and a mean that was used to calculate the second shape parameter: *μ* = *s*_1_/(*s*_1_ + *s*_2_). The shape parameters are *s* = 1 (*a*), *s* = ½ (*c*) and *s* = ⅓ (*e*). (*b*,*d*,*f*) Bimodal distributions that are also parametrized with a single shape parameter and a mean, describing the weighted average of two Beta distributions with fixed location means at the 10th and 90th percentile of the distribution (i.e. power = 0.145 and 0.905) for (*a,b*) (*s* = 1 and *s* = 2) and the 5th and 95th percentile (power = 0.0975 and 0.9525) for (*f*) (*s* = 1). Image (*c*) (*s* = ½) was used to model variance for the likely range, and alternative variances were modelled between (*a*) (small variance) and (*b*) (large variance). Extreme variance was modelled using the distribution in (*f*).

**Figure 2.**
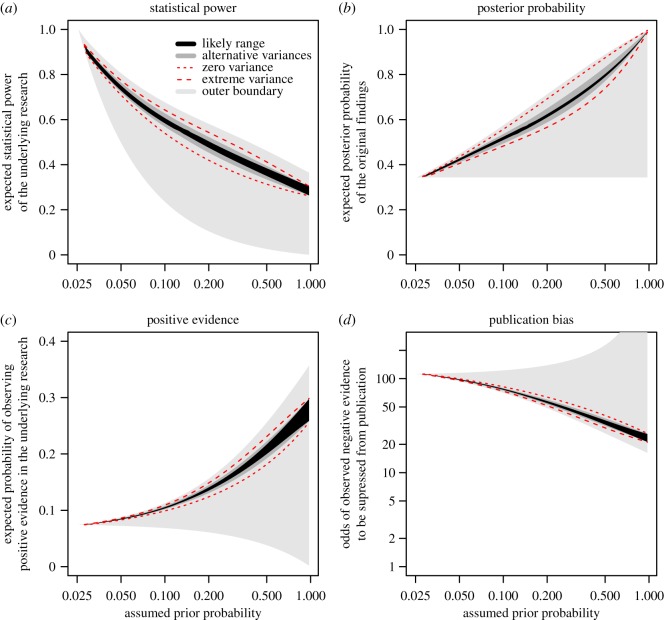
Expected statistical power and expected posterior probability of the original research replicated by the OSC (*a,b*) together with the expected proportion of observed positive evidence and the corresponding publication bias of the research (*c,d*), assuming a reproducibility rate of 36% and a literature with 90% positive evidence. The estimates were based on equations (6.1) and (6.2) for the range 0.025 < *θ* < 0.975 of assumed prior probabilities. The plots assume *α*_o_ = 0.05 in the original studies and *α*_r_ = 0.025 in the replication studies. The *likely range* assumes replication studies at 6–10% more statistical power than original studies, and that power in original studies followed a Beta distribution with shape parameter *s* = ½ (figure 1*c*). The *alternative variances* describe a range between a Beta distribution with shape parameter *s* = 1 (figure 1*a*) for smaller variance, and a bimodal distribution (figure 1*b*) for larger variance. The *extreme variance* estimate is based on a bimodal distribution (figure 1*f*) and the *zero variance* estimate is based on equation (5.1). *Outer boundaries* were calculated assuming anything from zero to extreme variance, and that statistical power in the replication studies fell between the power of the original studies and perfect power. *X*-axes of all plots and the *y*-axis of the publication bias plot (*d*) are on the log scale.

Statistical power is a function of the true effect size and the sample size of the study and does not have a well-defined sample distribution. Empirical studies based on a large number of meta-analyses indicate a bimodal distribution of power in the published literature, where a large proportion of studies have either very low or very high power [14,15]. We digitized the data on three research areas (somatic, psychiatric and neurological) presented by Dumas-Mallet *et al.* [14, figs 1 and 2] (see electronic supplementary material) and found that expected power was approximately in the range of 30–39% with variance 0.09–0.12. When only significant meta-analyses were considered, as an attempt to remove most true NULL associations, bimodality was reduced and expected power increased to about 42–51% with variance 0.08–0.11. We used these estimates as a starting point to find suitable distribution functions.

Figure 1 shows six distributions based on the Beta distribution function. The Beta distribution is defined by two shape parameters, and the mean is calculated as *μ* = *s*_1_/(*s*_1_ + *s*_2_). Figure 1*a*,*c*,*e* shows Beta distributions defined only by a single shape parameter (*s*), and the mean (*μ*) is used to calculate the second shape parameter. Panels in figure 1*b*,*d*,*f* are defined similarly, but describe bimodal distributions, calculated as the weighted average of two separate Beta distributions with fixed location means. The distribution that most closely matches the variances observed by Dumas-Mallet *et al*. is shown in figure 1*c* (*s* = ½), and we used it to model *likely* estimates. An *alternative variances* range was defined between a smaller variance defined in figure 1*a* and a larger variance in figure 1*b*. The distribution in figure 1*f* was used to model *extreme variance*.

## 7. Defining replication statistical power to solve the equations

In the discussion below, we use subscripts (*o* and *r*) to separate statistical power of the original studies (1 − *β*_o_) defined in equation (6.1) from power in replication studies (1 − *β*_r_) defined in equation (6.2).

The OSC determined the replication sample sizes from power analyses based on the reported effect sizes of the original studies. Such estimates are known to be inflated in the presence of publication bias [16] and cannot be used in our calculations. Data downloaded from the OSC GitHub repository [17] show that 70% of replications were designed with a larger sample than the original study, 10% had the same sample size and 20% were smaller than the original study, indicating that statistical power was on average higher in the replication studies. This information can be used to calculate upper and lower bounds of statistical power. The lower bound assumes identical power in original and replication studies, i.e. *β*_r_ = *β*_o_, and the upper bound assumes perfect power in replication studies *β*_r_ = 0. This reduces the number of unknowns to only two, and when power can be expected to be higher in the replication studies, it defines the boundaries of a range in which the true value *must* fall.

We can attempt a more precise approximation of power based on the median degrees of freedom of original studies (d.f. = 54) and replication studies (d.f. = 68) reported by the OSC. The observed median effect size in the replication studies (*r* = 0.2) is likely to be attenuated by the presence of NULL associations in data, and the observed effect size in the original studies (*r* = 0.4) is likely to be inflated by publication bias; thus, the true effect size is likely to fall between these two estimates. Calculating statistical power for the range 0.2 < *r* < 0.4 shows that a median-sized replication study added approximately 6–10% statistical power (10% at midpoint: *r* = 0.3) compared with the original study. This estimate gives an approximation of the increase in statistical power we can expect for the replication studies and allows for a likely range of power to be defined between *β*_r_ = *β*_0_ − 0.06 and *β*_r_ = *β*_0_ − 0.10. This gives two additional applications of equations (6.1) and (6.2) with only two unknown variables that define a range in which the true value is *likely* to fall.

## 8. Implications of observed reproducibility on prior probability of the tested hypotheses, statistical power of the studies, posterior probability of the original findings and publication bias

Assuming a true reproducibility rate of *R* = 0.36 (equations (3.3) and (6.2)) as reported by the OSC, a type 1 error rate of *α*_o_ = 0.05 for the original research (equations (3.2) and (6.1)), and because the OSC used two-tailed test of directional hypotheses, *α*_r_ = 0.025 in replication studies (equations (3.3) and (6.2)), together with the four different conditions of statistical power discussed above (*β*_r_ = *β*_o_, *β*_r_ = 0, *β*_r_ = *β*_o_ − 0.06 and *β*_r_ = *β*_o_ − 0.10), we have only two unknown variables left (*θ* and *β*_o_) and we can solve these equations to calculate the expected statistical power (1 − *μ*_β_) for any assumed prior probability (*θ*).

Equations (6.1) and (6.2) were solved as a system of two simultaneous equations using an optimizer, applying several different distributions of statistical power (figure 1). Equation (5.1) was solved analytically to represent the extreme boundary of zero variance in power. Solving these equations produced the expected statistical power of the research together with the corresponding expectation of the posterior probability of the original findings. We then applied equation (2.3) to calculate the expected probability of observing positive findings and compared that estimate with the approximately 90% positive findings that has been observed in the literature in order to assess publication bias. These results are summarized in figure 2 and the complete solution is presented in the electronic supplementary material.

The findings presented in figure 2 give insights into a plausible range of prior probabilities of tested hypotheses in psychology. Figure 2*a* shows that the prior probability of the underlying research was not likely to be *θ* < 0.025, because that would imply better than perfect expected power of the original research; and our suggested likely range, assuming +6–10% power in the replication studies, does not extend to *θ* < 0.027, because it would imply better than perfect power in the replications.

The prior was also unlikely to be smaller than *θ* < 0.05; while the lower bound of the power estimate at this prior fell at 50%, it is based on the implausible assumption of perfect power in the replications. The likely range suggests 73–75% expected power, which is quite optimistic, because such large statistical power has been indicated only for larger than medium effect sizes in psychological research [18,19]. A restricted range of priors was defined as 0.05 < *θ* < 0.20 that indicated expected power between 48 and 75%, and we assume this to be a plausible range in which the true prior is likely to fall.

Assuming smaller or larger *alternative variances* only marginally changed these estimates, to fall between 45 and 76% expected power. The range between zero and extreme variance brings expected power to 42–77%.

With higher assumed prior probabilities, the posterior probability of the original research goes up, and statistical power has to come down to be consistent with the reported reproducibility rate of 36%. Assuming that one out of 10 tested hypotheses in this research were true *a priori* (*θ* = 0.1), the posterior of the original findings was expected at 52% and the reason that the OSC could only replicate 36% is explained by 67% power in the replication studies. In addition, power in the original research was 59%, and 1.2% of the replications were expected to report type 1 errors.

For the full range of plausible priors 0.05 < *θ* < 0.20, the expected proportion of true-positive findings in the original studies fell between 42 and 62%. The alternative variances increased the range to 41–65% and assuming zero to extreme variance increased it further to 41–69%.

The most striking observation in figure 1 was the estimate of publication bias. Figure 1*e* indicates the expected proportion of positive evidence observed in the original studies to be between 8 and 14% for plausible priors; and this is also the distribution we would expect to observe in an unbiased literature. Assuming *extreme variance* brings this estimate up to a maximum of 15%. Figure 1*f* shows this estimate rescaled to odds of suppressing negative evidence in a literature with 90% positive evidence; even the lower bound of this estimate, above which the true estimate *must* fall if our assumptions hold, indicates that negative evidence was expected to be observed more than 16 times before one instance was published, over the whole range of priors plotted in figure 2. For the likely range and more plausible priors, 0.05 < *θ* < 0.20, we see an even more pronounced bias, indicating that negative evidence was likely to have been observed 55–98 times before one instance was published. Alternative variances suggest 53–99 times and the range between zero and extreme variance indicates 52–100. The lower end of the conservative outer bound fell in the range 49–94 for plausible priors.

## 9. Assuming reproducibility rates other than 36%

**Figure 3.**
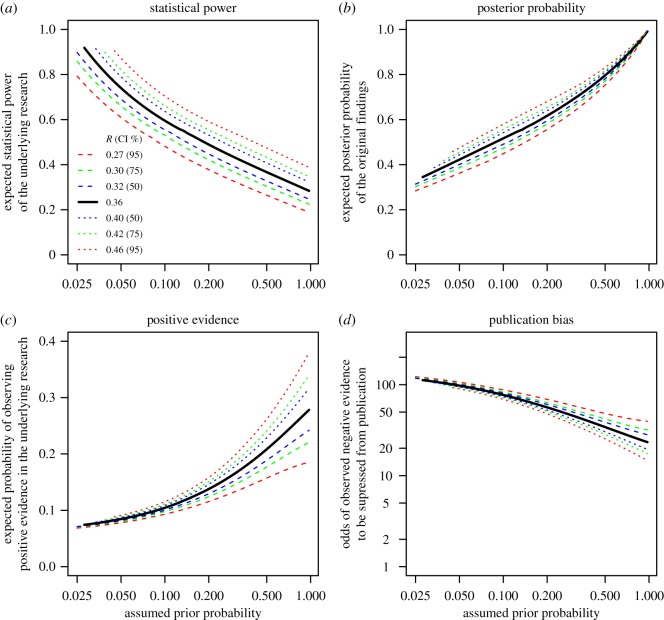
Expected statistical power and expected posterior probability of the original research replicated by the OSC (*a*,*b*) together with the expected proportion of observed positive evidence and the corresponding publication bias of the research (*c*,*d*), assuming different true replication rates and a literature with 90% positive evidence. The estimates were based on equations (6.1) and (6.2) assuming +8% power in the replication studies to reflect the midpoint of the likely range presented in figure 2. The variance of statistical power was also the same, assuming a Beta distribution with *s* = ½ (figure 1*c*). The lines describe the reported reproducibility rate (36%) and estimate at the outer limits of 50, 75 and 95% confidence intervals (i.e. *R* = 0.27, 0.30, 0.32, 0.36, 0.40, 0.42, 0.46).

So far, we have assumed the reproducibility rate to be 36%, which was the point estimate reported by the OSC. However, this is an estimate with uncertainty as indicated by the outer bounds of the 95% confidence intervals at 27 and 46% reproducibility. In figure 3, we expand our analysis to other plausible reproducibility rates, based on different confidence intervals of the OSC estimate. The analysis assumes +8% power in the replication studies to reflect the midpoint of our previous estimates and uses the same variance assumption as the likely range in figure 2. The different lines represent reproducibility rates at the outer bounds of the 50, 75 and 95% confidence intervals.

In general, assuming larger true reproducibility rates increased expected power and posterior probability of the research, but the expected proportion of positive evidence was only marginally affected. Assuming conservatively that the true reproducibility rate falls at the upper end of the 95% confidence interval (*R* = 46%), we expect to observe positive evidence 9–16% of the time for plausible priors (i.e. 0.05 < *θ* < 0.20), and the publication bias estimate indicates that negative evidence was suppressed 48–90 times before one instance was published, assuming a literature with 90% positive evidence.

## 10. Discussion

In this paper, we show how Bayes’ theorem can be used to better understand implications of the observed 36% reproducibility rate of published psychological findings that was reported by the OSC [1]. We demonstrated a method to assess publication bias and performed a tentative test indicating that the observed reproducibility rate was not consistent with an unbiased literature. We presented a mathematical solution and used it to estimate plausible ranges of *expected* statistical power, posterior probability, probability to observe positive evidence and publication bias of the underlying research.

We used Bayes’ theorem to calculate the expected (marginal) posterior probability assuming a known prior probability of the hypothesis, in order to solve a system of equations and find the expected statistical power needed to produce an expected reproducibility. Our solution produced the expectation after a large number of trials and does not allow for proper confidence (or credible) intervals to be computed. This differs from a full Bayesian model that makes explicit assumptions of prior distributions in order to estimate the posterior distribution of the parameters from the raw data [20] and reflects the limitations of using summary statistics for the analysis.

To perform these exercises, we made several assumptions: we assumed a prior probability that was independent of the statistical power of the studies testing the hypotheses; furthermore, we assumed that published research was reproducible 36% of the time [1], that replication studies had 6–10% better power than the original studies, that the variance in statistical power was similar to observations made in meta–meta-analyses [14] and that the literature presents 90% positive findings supporting the authors’ hypothesis [1–4]. The validity of our *likely* estimates depends on the validity of these assumptions. However, we also produced estimates for a range of plausible reproducibility rates and estimates based on *alternative variances.* In addition, we calculated *outer boundaries* that are valid for a range from *zero* to *extreme variance* and only assumed that the replication studies had higher power than the original studies.

The results showed that a long-term reproducibility rate of 36% is not consistent with a prior smaller than *θ* < 0.025, because it would imply better than perfect expected statistical power of the research. The prior was also unlikely to be smaller than *θ* < 0.05, because it would imply more than 73% expected power of the original research, which is an optimistic assumption. We suggest a plausible prior somewhere in the range 0.05 < *θ* < 0.20, indicating expected statistical power at 48–75%. We found that 42–62% of the original findings were expected to be true and that the reproducibility rate observed by the OSC was lower due to less than perfect power in the replications. Publication bias was large, assuming a literature with 90% positive findings, indicating that negative evidence was expected to be observed approximately 55–98 times before one negative result was published. Estimates of publication bias were robust and only marginally affected even assuming extreme variance, and assuming true replication rates up to 46%, representing the upper limit of the 95% confidence interval of the reproducibility estimate reported by the OSC.

Another analysis of the OSC data by Johnson *et al*. [20] focused on observed effect sizes and was restricted to the subsample for which a correlation (*r*) with standard errors could be derived (73/100 studies). This subsample had 71 positive findings and the observed reproducibility rate was 41%. The authors estimated approximately 93% true NULL hypotheses in this research, i.e. *θ* = 0.07. Furthermore, they estimated *α*_o_ = 0.052 and that both original studies and replication studies had 75% power to arrive at an estimated posterior of 37/71 = 52% of the original positive findings. They also indicated that approximately 700 hypothesis tests were performed to produce the 71 positive and two negative published findings in the sample, suggesting that more than 600 negative findings had been observed in the process.

Our analysis was based on the observed reproducibility (36%) for the full sample of positive findings replicated by the OSC. Also, because the replications were designed with larger sample sizes in average, we did not assume identical power in the replication studies and original studies and used that assumption only for the outer boundary. If we were to accept the prior suggested by Johnson *et al*. [20] (i.e. *θ* = 0.07), expected statistical power would be estimated in the range 65–67% in the original studies and 73–75% in the replication studies. The expected posterior of the original findings would be 46–47%. In addition, the expected proportion of positive evidence observed in the original studies was approximately 9.3%, suggesting that 97/0.093 = 1043 studies were needed to produce the 97 positive and three negative findings that were published and subsequently replicated by the OSC; this means that negative evidence was observed approximately 88 times before a negative finding was published, assuming a literature with 90% positive findings.

The prior (*θ* = 0.07) suggested by Johnson *et al.* [20] implies more than 65% expected power of the original research. Such high power has been indicated for larger than medium (*r* = 0.3) effect sizes in psychological research [18,19] and is larger than empirical estimates of median power observed in other fields [14,15]. Considering that the median effect size observed in the replication studies by OSC was only *r* = 0.2, assuming such high power is optimistic, but not implausible due to the likely attenuation of this estimate from the presence of NULL associations in data. We propose a plausible prior somewhere in the range 0.05 < *θ* < 0.20, corresponding to expected statistical power in the range 48–75% of the original studies. However, for completeness we presented results for the full range of assumed priors, so that readers can investigate the implications of assumptions that fall outside of our suggested range.

Applying Bayes’ theorem in this way has important implications: it assumes that hypotheses are either true or false, and such binary hypothesis testing has been criticized [21]. Indeed, it can be argued that there are no truly non-zero associations in *observational* data. If we assume that no associations are truly zero, but we are not interested in making inferences from very small true effect sizes, *p*-values from NULL hypothesis significance testing (NHST) would be biased with inflated type 1 errors. In addition, we may conclude that any (non-directional) hypothesis is necessarily true, giving a trivial prior probability of *θ* = 1. However, we should recognize that these are not limitations of binary hypothesis testing *per se*, but rather limitations of how specific hypotheses are formulated and tested. It is possible to define a different ‘NULL’ hypothesis, with a mean other than zero, to protect inferences from true effect sizes of ‘trivial’ magnitudes [22] and make the prior more informative in observational studies at *θ* < 1. Also, binary NHST is not inherently problematic in *true* experimental designs (with randomization), because we can then assume associations in data that are truly NULL. In the present analysis, we have assumed the same position on binary NHST as the publishing authors of the original studies that were replicated by the OSC, and the limitations discussed above apply similarly to how they would apply to the original studies.

The most crucial estimate used in our analysis was the observed reproducibility rate of 36% reported by the OSC [1]. Reproducibility is a complicated concept with many different facets, in particular in psychology and the social sciences; some ‘true’ findings may not be possible to replicate in a different time, social or cultural context, because the underlying meaning of the constructs used to design the study or define the variables may have changed. The underlying theory may still be valid but needs to be adapted to the new environment, and this has been proposed as an argument against the validity of direct replication of a study's methods on an independent sample [23]. But, from a more general scientific perspective, it can be seen as a flaw in the formulated theory and the methods defined to test it: Science needs to be verifiable to stand out from other types of claims and should have some generalizability to be a useful source of knowledge; thus, important context needs to be included when formulating a scientific theory or hypothesis. Another factor to consider is poorly described methods in the original study that may impact the success rate in replications, but this is essentially the same problem. If the study report did not present sufficient information to accurately replicate the methods: how can it be properly understood and evaluated by the readers?

Reproducibility may have been impaired because of mistakes made by the replicating team of researchers; however, this does not seem to be a major risk in the OSC study. The study was pre-registered and performed by well-motivated researchers under more or less public scrutiny; the team was in frequent contact with authors of the original studies to obtain material and information about the design and procedure of their studies; and they employed a system of internal reviews of all studies to ensure quality. Our findings show that assuming a larger true reproducibility rate of this research implies larger statistical power and posterior probability of the original findings, but estimates of publication bias were only marginally affected. In addition, any potential mistakes that may have lowered the reproducibility rate below its true value are part of the overall type 2 error rate (*β*) in equations (3.3) and (6.2) and can be seen as a reduction of ‘statistical power’ in the replication studies below what we have nominally assumed. It seems unlikely that this would pose a problem large enough to invalidate the lower bound of the estimate used in this study, assuming power to be identical in the original and replication studies.

Studies eligible for replication by the OSC were selected from three prestigious journals in experimental psychology. Approximately one-third of the total sample was never submitted for replication, mostly because these studies were deemed infeasible to replicate, for example, because they required special samples, knowledge or equipment. This introduces uncertainty and potential bias in the reproducibility estimate; it is possible that the more specialized or complicated designs would have worse (or, less likely, better) reproducibility. Thus, the reproducibility rate estimated by the OSC is an estimate representative of the two-thirds most accessible research in three well-regarded journals in experimental psychology; and might not generalize to psychology in general.

Data from other scientific fields suggest a less pronounced focus on positive evidence, with 70–90% significant findings supporting the authors' hypothesis [3,4], but even worse reproducibility rates in the range 11–24% in certain fields [24,25]. This suggests that while all estimates presented here may not generalize, publication bias may still be of similar magnitude in other fields; but specific fields with a higher proportion of published negative evidence and, to some extent, with a higher demonstrated reproducibility [26] are likely to be less affected by publication bias.

One should recognize that most findings suppressed from publication describe NULL effects that many may find uninformative or not interesting [23], but the fact that they are never published makes it more likely that similar studies are performed repeatedly by independent researchers; and eventually one will become ‘significant’ by chance, dramatically increasing its chance of being published. Thus, the fact that such a large portion of negative evidence was suppressed from publication not only represents a serious threat to the veracity of published positive evidence; it also means that false theories that have been published may never become ‘falsified’ in the literature [5] and that researchers are likely to spend time and resources testing hypotheses that should already have been rejected.

We estimated the expected magnitude of total bias related to publishing findings in psychological journals. This bias is produced at many different stages in the research process, and we cannot say how much is related to editorial decisions to reject publication, researchers putting negative findings in the file drawer [6], selective reporting, HARKing [7] or *p*-hacking [8–10]. Our metric assumes independent observations; however, in the case of repeated observations in a single study, we expect observations to be correlated. Thus, our estimate would tend to be conservative with respect to actual observations made in data, because correlated observations provide less new information than independent observations.

Publication bias may be the single most important problem to solve in order to increase the efficiency of the scientific project and bring the veracity of published research to higher standards. The implications of suppressing more than 55 negative observations for each one published should not be underestimated. With *α* = 0.05, we expect a significant finding by chance for every 20 observations made on random data. Thus, our results suggest that even when studied associations are truly NULL, the literature will be dominated by statistically significant findings.

## Supplementary Material

Supplementary information

## Supplementary Material

Source code to reproduce all findings

## Data accessibility

All the code needed to reproduce the findings presented in this paper is available as an electronic supplementary material, appendix.

## References

[1] Open Science Collaboration. 2015 Estimating the reproducibility of psychological science. Science 349, aac4716 (10.1126/science.aac4716) 26315443

[2] Sterling TD 1959 Publication decisions and their possible effects on inferences drawn from tests of significance—or vice versa. J. Am. Stat. Assoc. 54, 30–34.

[3] Sterling TD, Rosenbaum WL, Weinkam JJ 1995 Publication decisions revisited: the effect of the outcome of statistical tests on the decision to publish and vice versa. Am. Stat. 49, 108–112.

[4] Fanelli D 2010 ‘Positive’ results increase down the hierarchy of the sciences. PLoS ONE 5, e10068 (10.1371/journal.pone.0010068) 20383332 PMC2850928

[5] Ferguson CJ, Heene M 2012 A vast graveyard of undead theories: publication bias and psychological science's aversion to the null. Perspect. Psychol. Sci. 7, 555–561. (10.1177/1745691612459059) 26168112

[6] Rosenthal R 1979 The file drawer problem and tolerance for null results. Psychol. Bull. 86, 638 (10.1037/0033-2909.86.3.638)

[7] Kerr NL 1998 HARKing: hypothesizing after the results are known. Pers. Soc. Psychol. Rev. 2, 196–217. (10.1207/s15327957pspr0203_4) 15647155

[8] Head ML, Holman L, Lanfear R, Kahn AT, Jennions MD 2015 The extent and consequences of p-hacking in science. PLoS Biol. 13, e1002106 (10.1371/journal.pbio.1002106) 25768323 PMC4359000

[9] Bruns SB, Ioannidis JPA 2016 p-Curve and p-hacking in observational research. PLoS ONE 11, e0149144 (10.1371/journal.pone.0149144) 26886098 PMC4757561

[10] Simmons JP, Nelson LD, Simonsohn U 2011 False-positive psychology: undisclosed flexibility in data collection and analysis allows presenting anything as significant. Psychol. Sci. 22, 1359–1366. (10.1177/0956797611417632) 22006061

[11] R Core Team. 2017 *R: a language and environment for statistical computing* Vienna, Austria: R Foundation for Statistical Computing See https://www.R-project.org/.

[12] Puga JL, Krzywinski M, Altman N 2015 Bayes' theorem. Nat. Methods 12, 277 (10.1038/nmeth.3335) 26005726

[13] Dreber A, Pfeiffer T, Almenberg J, Isaksson S, Wilson B, Chen Y, Nosek BA, Johannesson M 2015 Using prediction markets to estimate the reproducibility of scientific research. Proc. Natl Acad. Sci. USA 112, 15 343–15 347. (10.1073/pnas.1516179112) PMC4687569 26553988

[14] Dumas-Mallet E, Button KS, Boraud T, Gonon F, Munafò MR 2017 Low statistical power in biomedical science: a review of three human research domains. R. Soc. open sci. 4, 160254 (10.1098/rsos.160254) 28386409 PMC5367316

[15] Button KS, Ioannidis JPA, Mokrysz C, Nosek BA, Flint J, Robinson ESJ, Munafò MR 2013 Power failure: why small sample size undermines the reliability of neuroscience. Nat. Rev. Neurosci. 14, 365–376. (10.1038/nrn3475) 23571845

[16] Yarkoni T 2009 Big correlations in little studies: inflated fMRI correlations reflect low statistical power: commentary on Vul *et al*. (2009). Perspect. Psychol. Sci. 4, 294–298. (10.1111/j.1745-6924.2009.01127.x) 26158966

[17] (2017). https://github.com/CenterForOpenScience/rpp.

[18] Szucs D, Ioannidis JPA 2017 Empirical assessment of published effect sizes and power in the recent cognitive neuroscience and psychology literature. PLoS Biol. 15, e2000797 (10.1371/journal.pbio.2000797) 28253258 PMC5333800

[19] Rossi JS 1990 Statistical power of psychological research: what have we gained in 20 years? J. Consult. Clin. Psychol. 58, 646 (10.1037/0022-006X.58.5.646) 2254513

[20] Johnson VE, Payne RD, Wang T, Asher A, Mandal S 2017 On the reproducibility of psychological science. J. Am. Stat. Assoc. 112, 1–10. (10.1080/01621459.2016.1240079) 29861517 PMC5976261

[21] Cohen J 1994 The earth is round (p<0.05). Am. Psychol. 49, 997 (10.1037/0003-066X.49.12.997)

[22] Ingre M 2013 Why small low-powered studies are worse than large high-powered studies and how to protect against ‘trivial’ findings in research: comment on Friston (2012). Neuroimage 81, 496–498. (10.1016/j.neuroimage.2013.03.030) 23583358

[23] Stroebe W, Strack F 2014 The alleged crisis and the illusion of exact replication. Perspect. Psychol. Sci. 9, 59–71. (10.1177/1745691613514450) 26173241

[24] Prinz F, Schlange T, Asadullah K 2011 Believe it or not: how much can we rely on published data on potential drug targets? Nat. Rev. Drug Discov. 10, 712 (10.1038/nrd3439-c1) 21892149

[25] Begley CG, Ellis LM 2012 Drug development: raise standards for preclinical cancer research. Nature 483, 531–533. (10.1038/483531a) 22460880

[26] Camerer CF et al. 2016 Evaluating replicability of laboratory experiments in economics. Science 351, 1433–1436. (10.1126/science.aaf0918) 26940865

