# Supplementary material for: Estimating statistical power, posterior probability and publication bias of psychological research using the observed replication rate
Source: R Soc Open Sci. 2018 Sep 12;5(9):181190. doi: 10.1098/rsos.181190 (PMC6170554; doi:10.1098/rsos.181190)
Supplement: Supplementary information [file rsos181190supp1.pdf]

Supplemental material

Power in meta-meta analysis by Dumas-Mallet et al

Table S1 show power distributions extracted from figure 1 and 2 by Dumas-Mallet et al (2017).

Table S1: Power distributions in three research fields extracted from Dumas-Mallet et al (2017)

Power	Somatic	Psychiatric	Neurological
	All meta-analyses		
0.05	0.376	0.302	0.368
0.15	0.201	0.128	0.146
0.25	0.095	0.103	0.081
0.35	0.043	0.071	0.057
0.45	0.043	0.068	0.030
0.55	0.050	0.052	0.062
0.65	0.027	0.037	0.043
0.75	0.027	0.030	0.043
0.85	0.060	0.021	0.037
0.95	0.078	0.188	0.132
Mean	0.297	0.387	0.346
Variance	0.092	0.116	0.110
	Statistically significant meta-analyses		
0.05	0.105	0.089	0.095
0.15	0.232	0.187	0.148
0.25	0.125	0.187	0.138
0.35	0.057	0.103	0.073
0.45	0.067	0.103	0.052
0.55	0.077	0.103	0.074
0.65	0.047	0.044	0.073
0.75	0.047	0.025	0.063
0.85	0.106	0.018	0.063
0.95	0.136	0.142	0.223
Mean	0.449	0.510	0.415
Variance	0.101	0.082	0.106

Solving equation 6 to find β_o from θ

The system of equations defined by equation 6 in the main text (replicated below with subscripts *o* for quantities related to original studies and *r* for replication studies) needs to be solved for unique values of the reproducibility rate (R), the assumed type-2 error rate in the replication studies (β_r) as well as the type-1 error rate of the original (α_o) and replication studies (α_r). This can be simplified using a computerized equation solver and cross checking the math of the suggested solution. Syntax for solving the equations using a web based equation solver (www.wolframalpha.com) together with R-code for cross checking the math is given below.

$$\begin{cases} \hat{\theta} = \frac{\theta(1-\beta_o)}{\theta(1-\beta_o)+\alpha_o(1-\theta)} \\ R = \hat{\theta}(1-\beta_r) + \alpha_r(1-\hat{\theta}) \end{cases} \qquad \text{Eq (S1)}$$

Since the picked equation solver was somewhat limited in the choice of symbols, equation S1 was rewritten in plain text like so:

```
P=(theta*(1-beta))/(theta*(1-beta)+alpha*(1-theta));
R=P*(1-b)+a*(1-P);
```

To find the solution for the lower bound of the range, in which the true value must fall (discussed in detail in the main text), we assume identical power in the original and replication studies, and added the following constraints:

```
alpha=.05; R=.36; a=.025; b=beta; 0<theta<1;
```

And to finish the command we added instructions to solve for β_o and $\hat{\theta}$:

```
solve beta and P
```

This produced the following solution for the lower bound of the range in which the true value *must* fall (see the main text):

$$\beta = b \text{ and } P = \frac{20(\beta-1)\theta}{(20\beta-19)\theta-1} \text{ and } 0 < \theta < 1 \text{ and } b = \frac{1}{200}\left(164 - \sqrt{\frac{670}{\theta} + 626}\right)$$

```
P = (theta*(1-beta))/
    (theta*(1-beta)+alpha*(1-theta));
R = P*(1-b)+a*(1-P); alpha = .05;
R = .36; a = .025; b = beta;
0 < theta < 1; solve beta and P
```

Computed by Wolfram|Alpha

Solving the equation for the upper bound of this range produced the following solution:

$$\beta = \frac{2627\,\theta - 67}{2560\,\theta} \quad \text{and} \quad P = \frac{67}{195} \quad \text{and} \quad 0 < \theta < 1$$

```
P = (theta * (1 - beta)) /
    (theta * (1 - beta) + alpha * (1 - theta));
R = P * (1 - b) + a * (1 - P);  alpha = .05;
R = .36;  a = .025;  b = 0;
0 < theta < 1;  solve beta and P
```

Computed by Wolfram|Alpha

Solving the equation for the lower end of the more narrow *likely* range (see discussion in the main text) produced the following solution (note, this solution is valid for the whole range $0 < \theta < 1$ but the solver erroneously produced a constant solution for the specific case of $\theta = 10/17$):

$$\beta = b + \frac{3}{50} \quad \text{and} \quad P = \frac{20\,(\beta - 1)\,\theta}{(20\,\beta - 19)\,\theta - 1} \quad \text{and}$$
$$0 < \theta < \frac{10}{17} \quad \text{and} \quad b = \frac{1}{200}\left(158 - \sqrt{\frac{670}{\theta} + 230}\right)$$

```
P =
  (theta * (1 - beta)) / (theta * (1 - beta) + alpha * (1 - theta));
R = P * (1 - b) + a * (1 - P);  alpha = .05;
R = .36;  a = .025;  b = beta - 0.06;
0 < theta < 1;  solve beta and P
```

Computed by Wolfram|Alpha

Solving the equation for the upper end of the *likely* range produced the following solution (note, similar to above we got an erroneous constant solution for $\theta = 2/5$):

$$\beta = b + \frac{1}{10} \quad \text{and} \quad P = \frac{20\,(\beta - 1)\,\theta}{(20\,\beta - 19)\,\theta - 1} \quad \text{and} \quad 0 < \theta < \frac{2}{5} \quad \text{and} \quad b = \frac{1}{200}\left(154 - \sqrt{\frac{670}{\theta} + 6}\right)$$

```
P = (theta * (1 - beta)) / (theta * (1 - beta) + alpha * (1 - theta));
R = P * (1 - b) + a * (1 - P);  alpha = .05;  R = .36;  a = .025;
b = beta - 0.10;  0 < theta < 1;  solve beta and P
```

Computed by Wolfram|Alpha

Summary of estimates for all distributions presented in figure 1

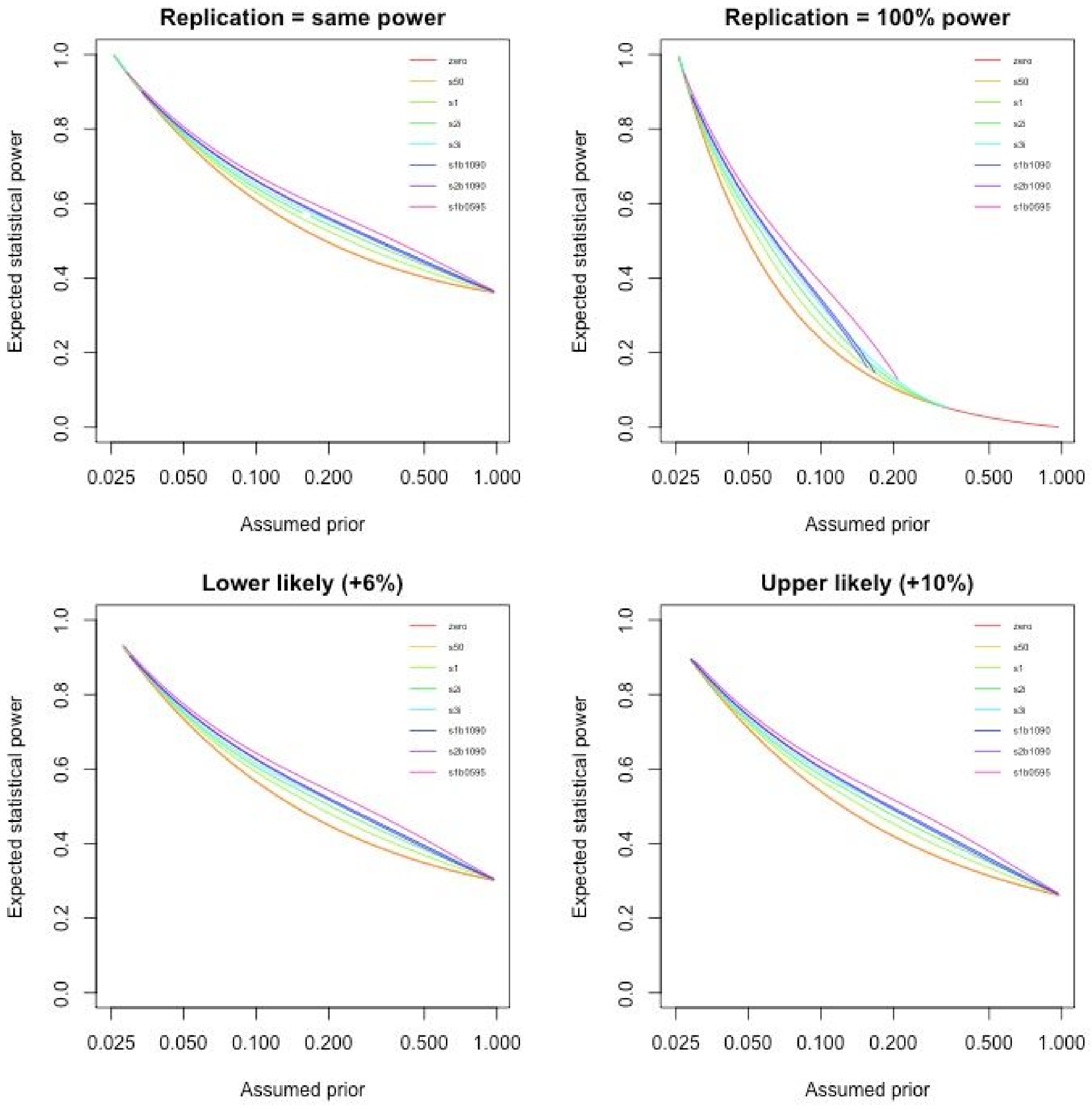

Figure S1: Expected statistical power for the four conditions. The top two panels describe the outer bounds and the bottom two describe the limits of the likely interval. Estimates are presented for the naive analytical assuming zero variance in power (zero) and for Beta distributions with shape parameter s50-s3i (s=50, 1, 1/2, 1/3) and for bimodal distributions with location means at 10/90th percentiles of the distribution with s=1 (s1b1090) and s=2 (s2b1090) and at 05/95th percentiles with s=1 (s1b0595).

-

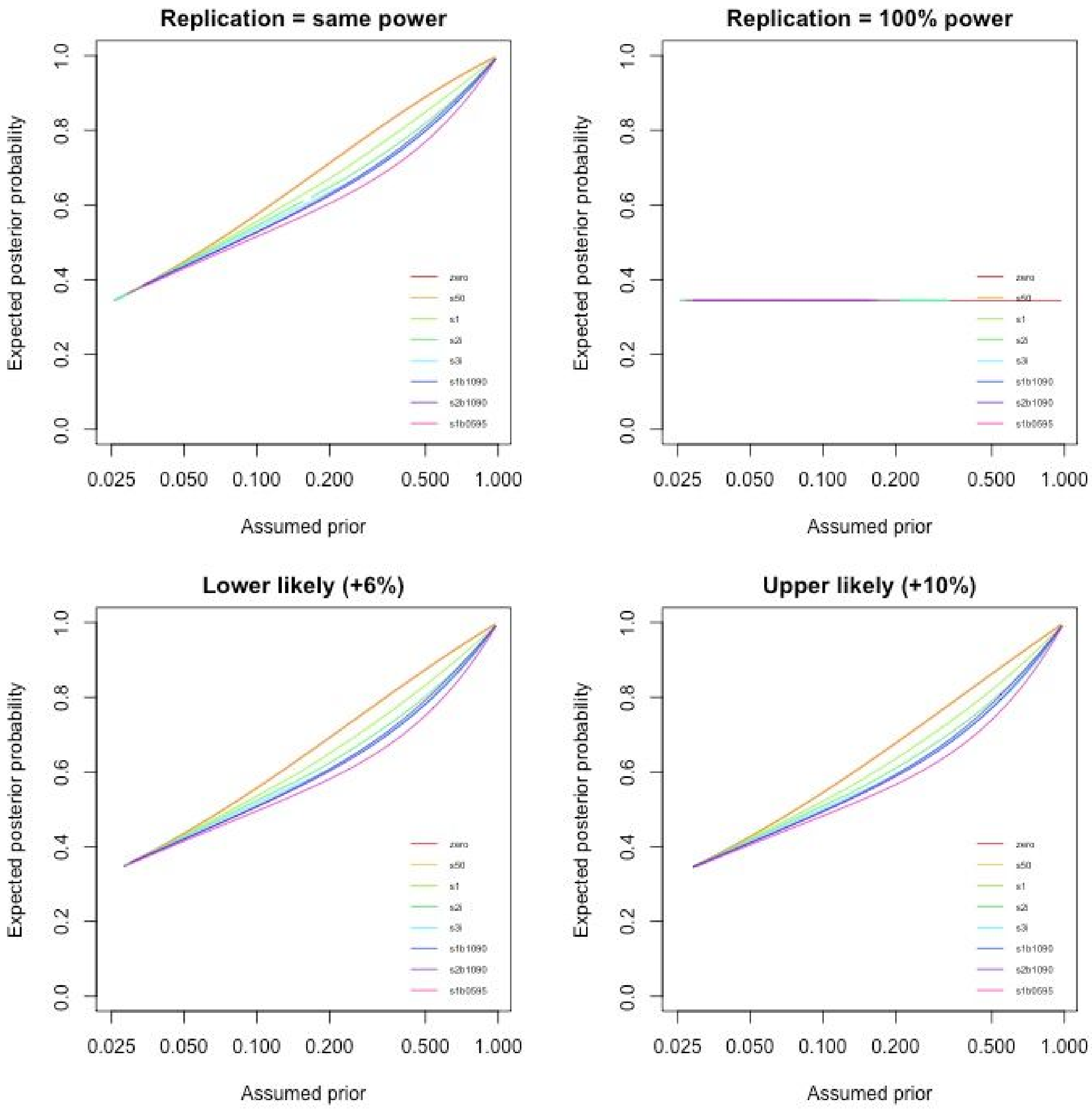

Figure S2: Expected posterior probability for the four conditions. The top two panels describe the outer bounds and the bottom two describe the limits of the likely interval. Estimates are presented for the naive analytical assuming zero variance in power (zero) and for Beta distributions with shape parameter s50-s3i (s=50, 1, 1/2, 1/3) and for bimodal distributions with location means at 10/90th percentiles of the distribution with s=1 (s1b1090) and s=2 (s2b1090) and at 05/95th percentiles with s=1 (s1b0595).

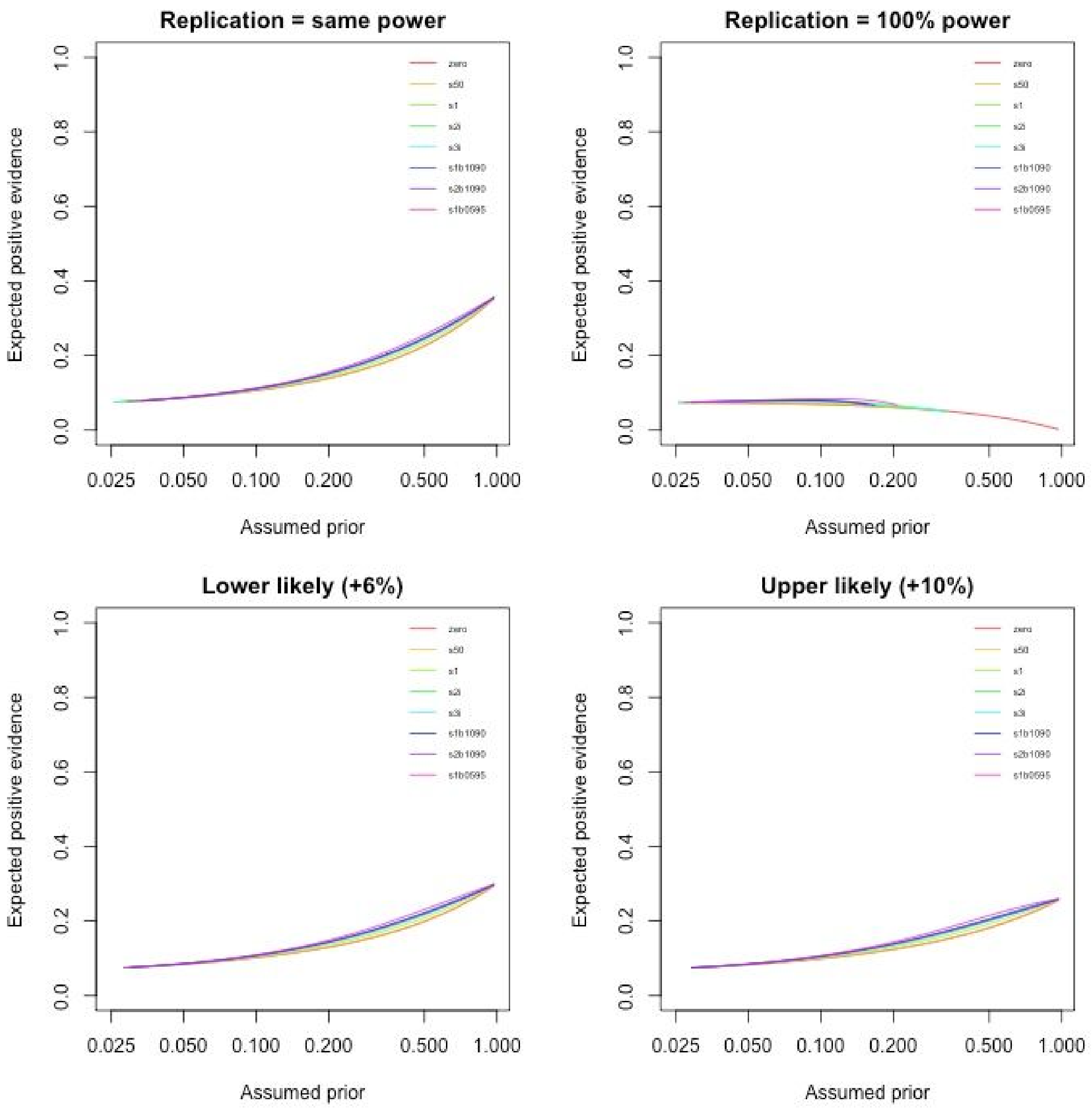

Figure S3: Expected positive evidence for the four conditions. The top two panels describe the outer bounds and the bottom two describe the limits of the likely interval. Estimates are presented for the naive analytical assuming zero variance in power (zero) and for Beta distributions with shape parameter s50-s3i (s=50, 1, 1/2, 1/3) and for bimodal distributions with location means at 10/90th percentiles of the distribution with s=1 (s1b1090) and s=2 (s2b1090) and at 05/95th percentiles with s=1 (s1b0595).

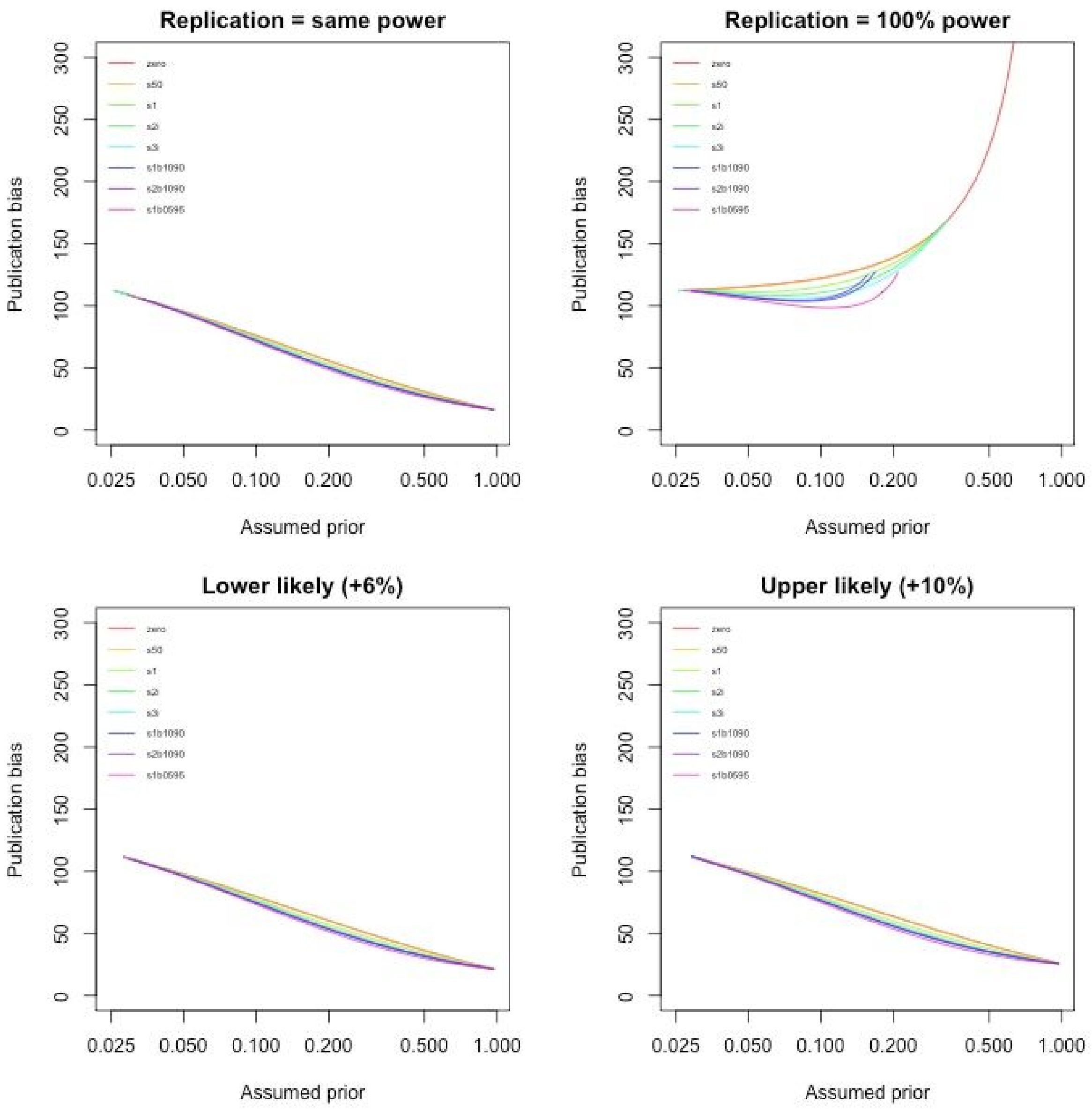

Figure S1: Expected publication bias for the four conditions. The top two panels describe the outer bounds and the bottom two describe the limits of the likely interval. Estimates are presented for the naive analytical assuming zero variance in power (zero) and for Beta distributions with shape parameter s50-s3i (s=50, 1, 1/2, 1/3) and for bimodal distributions with location means at 10/90th percentiles of the distribution with s=1 (s1b1090) and s=2 (s2b1090) and at 05/95th percentiles with s=1 (s1b0595).

R-code to reproduce all findings presented in the paper

The R-code needed to reproduce all findings in this paper is attached as a separate zip archive. The code can also be downloaded here:
https://github.com/micing/publication_bias_psychology
