# Supplementary figures and images for: Estimating statistical power, posterior probability and publication bias of psychological research using the observed replication rate
Source: R Soc Open Sci. 2018 Sep 12;5(9):181190. doi: 10.1098/rsos.181190 (PMC6170554; doi:10.1098/rsos.181190)

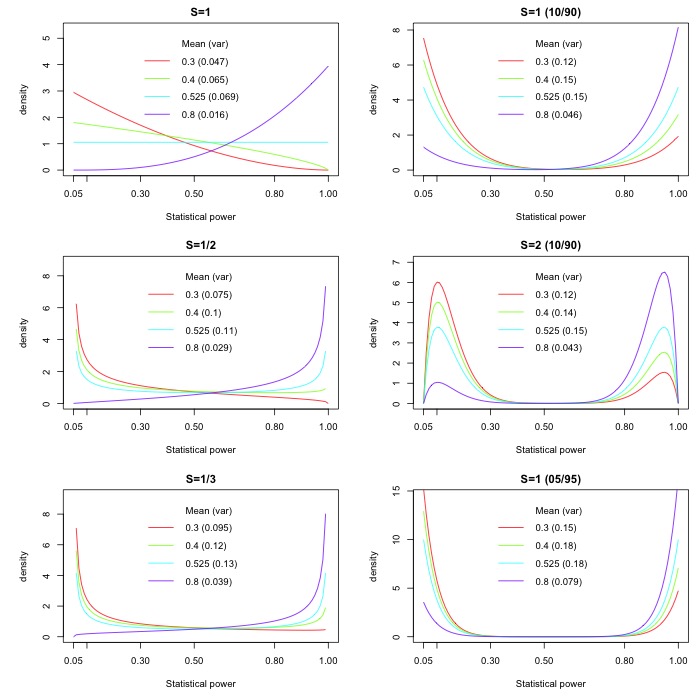

Supplement: Source code to reproduce all findings [file rsos181190supp2.zip › publication_bias_psychology-master/jpeg/figure1.jpg]

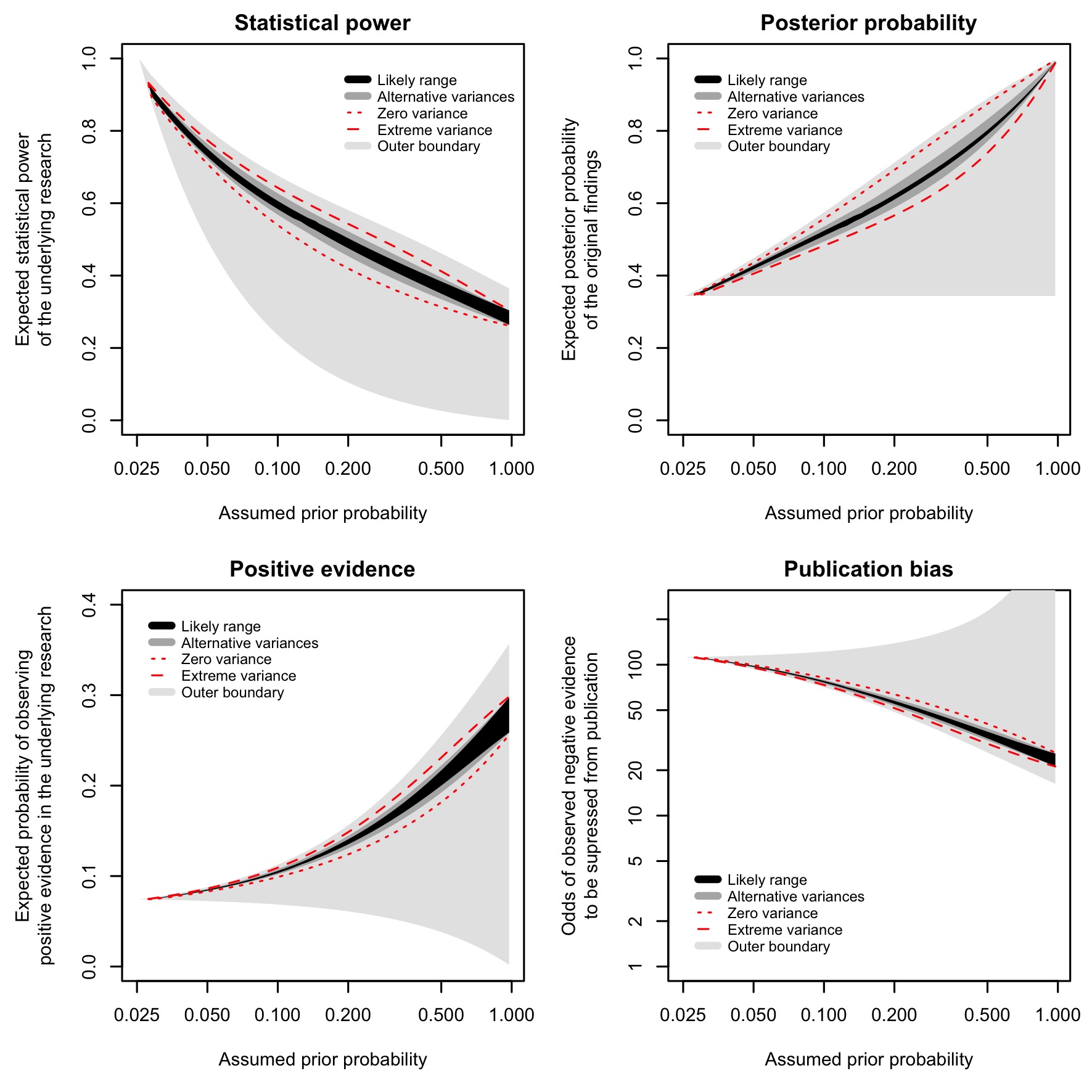

Supplement: Source code to reproduce all findings [file rsos181190supp2.zip › publication_bias_psychology-master/jpeg/figure2.jpg]

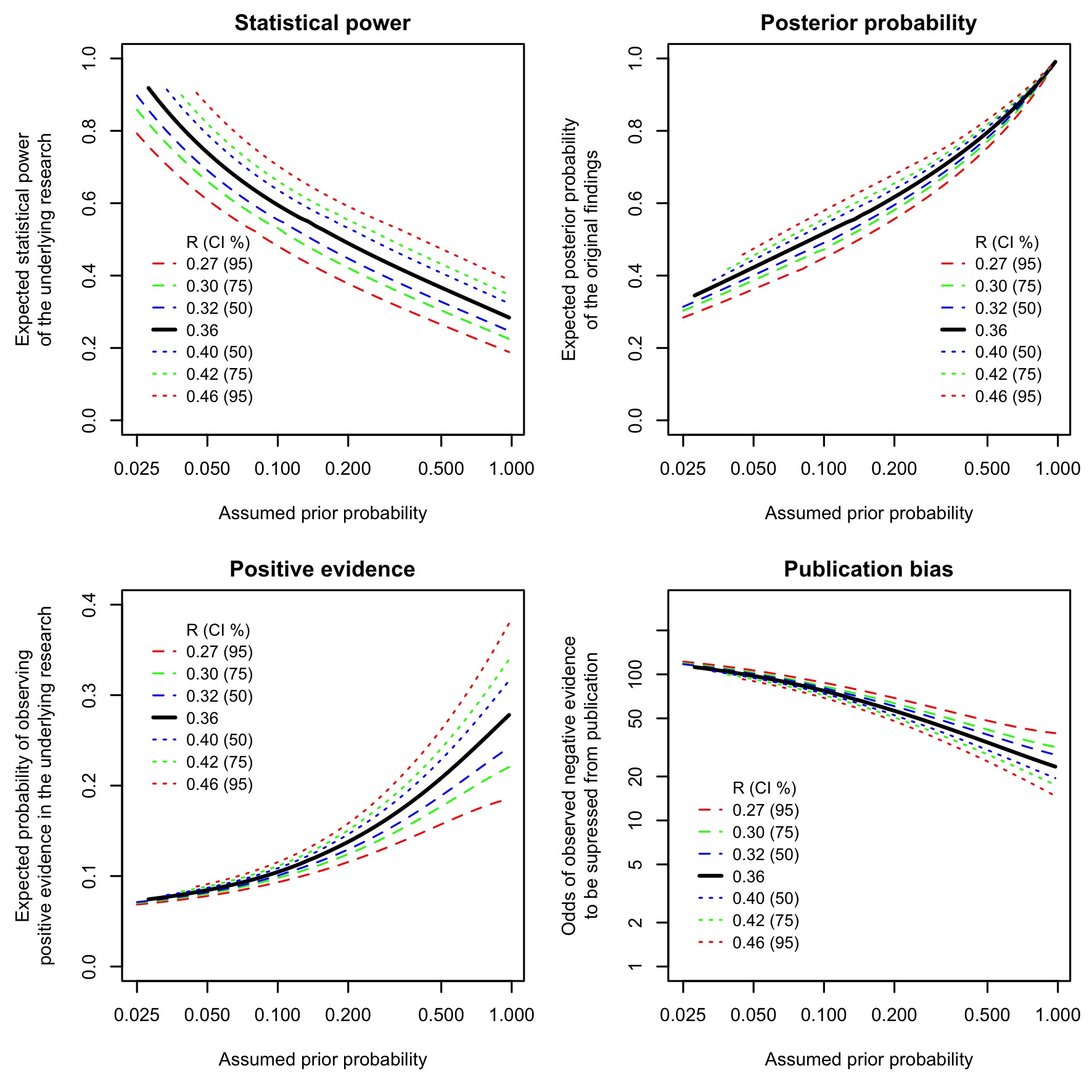

Supplement: Source code to reproduce all findings [file rsos181190supp2.zip › publication_bias_psychology-master/jpeg/figure3.jpg]

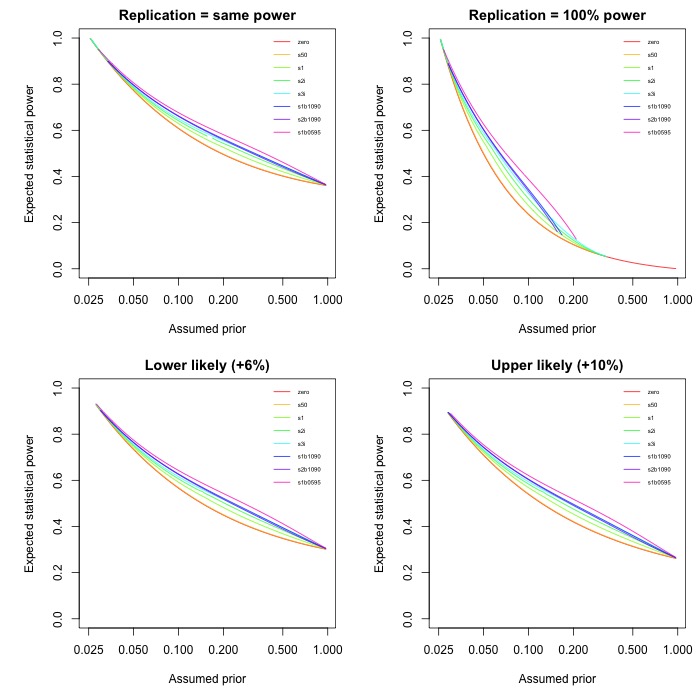

Supplement: Source code to reproduce all findings [file rsos181190supp2.zip › publication_bias_psychology-master/jpeg/figureS1.jpg]

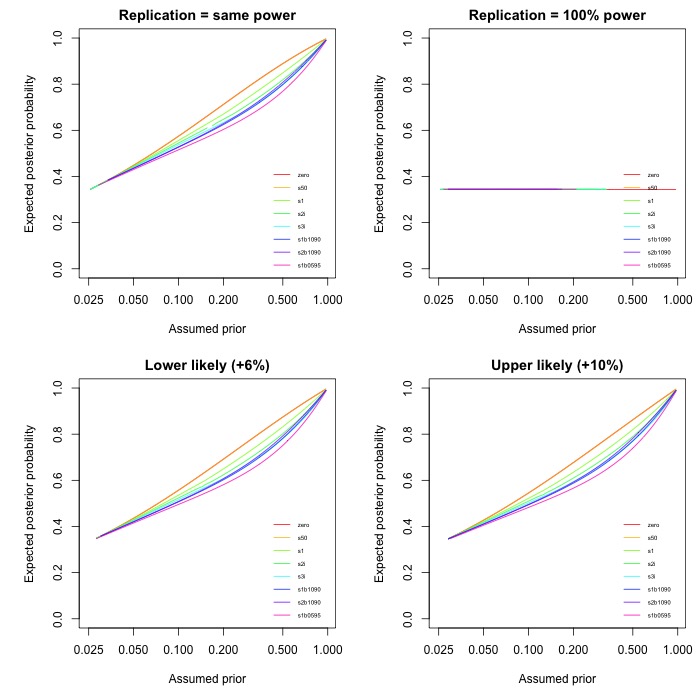

Supplement: Source code to reproduce all findings [file rsos181190supp2.zip › publication_bias_psychology-master/jpeg/figureS2.jpg]

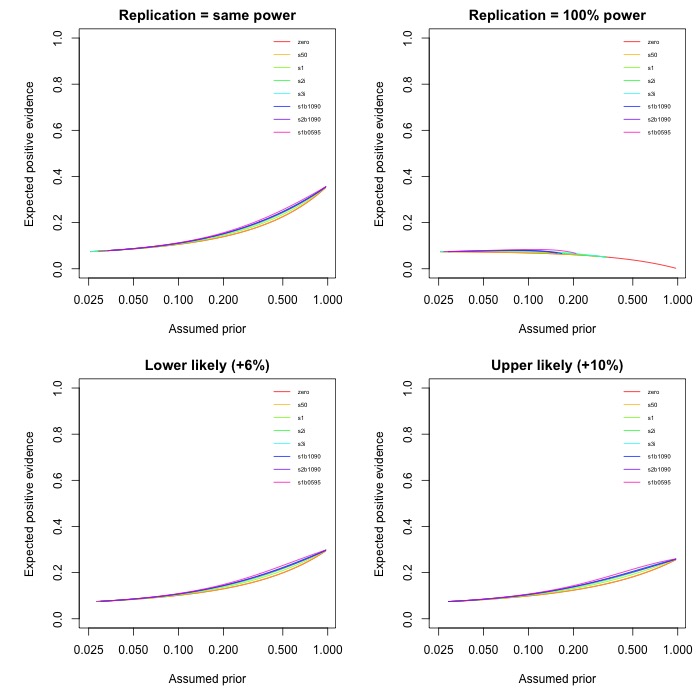

Supplement: Source code to reproduce all findings [file rsos181190supp2.zip › publication_bias_psychology-master/jpeg/figureS3.jpg]

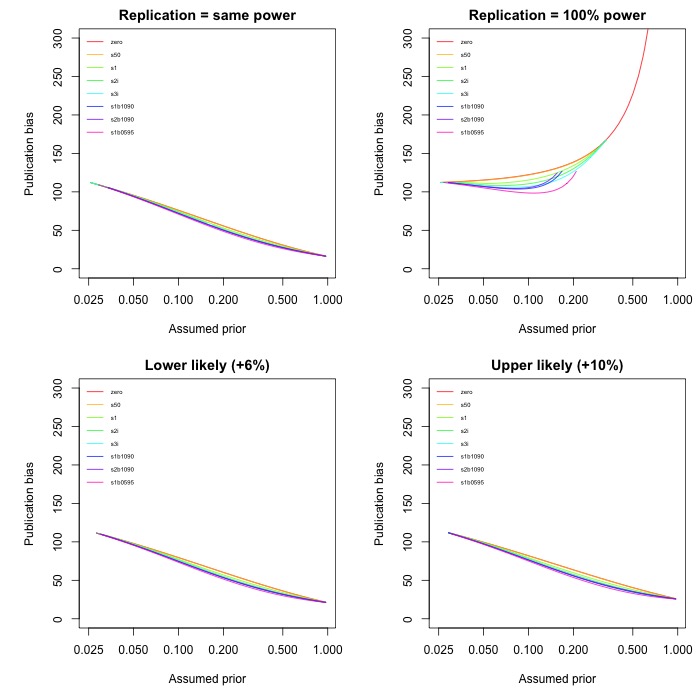

Supplement: Source code to reproduce all findings [file rsos181190supp2.zip › publication_bias_psychology-master/jpeg/figureS4.jpg]
